# The estimated burden of ultra-processed foods on cardiovascular disease outcomes in Brazil: A modeling study

**DOI:** 10.3389/fnut.2022.1043620

**Published:** 2022-11-17

**Authors:** Eduardo Augusto Fernandes Nilson, Gerson Ferrari, Maria Laura da Costa Louzada, Renata Bertazzi Levy, Carlos Augusto Monteiro, Leandro F. M. Rezende

**Affiliations:** ^1^Center for Epidemiological Research in Nutrition and Public Health, University of São Paulo, São Paulo, Brazil; ^2^Oswaldo Cruz Foundation-Fiocruz, Brasília, Brazil; ^3^Escuela de Ciencias de la Actividad Física, el Deporte y la Salud, Universidad de Santiago de Chile (USACH), Santiago, Chile; ^4^Facultad de Ciencias de la Salud, Universidad Autónoma de Chile, Santiago, Chile; ^5^Departamento de Nutrição, Faculdade de Saúde Pública, Universidade de São Paulo, São Paulo, Brazil; ^6^Department of Preventive Medicine, Faculdade de Medicina FMUSP, Universidade de São Paulo, São Paulo, Brazil; ^7^Department of Preventive Medicine, Escola Paulista de Medicina, Universidade Federal de São Paulo, São Paulo, Brazil

**Keywords:** ultra-processed foods, CVD, mortality, incidence, DALYs, modeling

## Abstract

**Introduction:**

Ultra-processed foods (UPF) have been associated with an increased risk of cardiovascular diseases (CVD). This study aimed to estimate CVD premature deaths, incident cases, and disability adjusted life-years (DALYs) attributable to the consumption of UPF in Brazilian adults in 2019.

**Methods:**

A validated a comparative risk assessment model was adapted to estimate the burden of major CVD outcomes (coronary heart disease and stroke) attributable to the consumption of UPF in Brazilian adults aged 30 to 69 years. The model inputs included nationally representative data of the UPF contribution to the total energy of the diet, national official demographic records, CVD outcomes (incidence, deaths and DALYs) from the Global Burden of Disease study for 2019, and relative risks from meta-analysis studies.

**Results:**

We estimated that approximately 19,200 premature deaths (95% uncertainty intervals – UI, 7,097 to 32,353), 74,900 new cases (95% UI, 25,983 to 128,725), and 883,000 DALYs/year (95% UI, 324,279 to 1,492,593) from CVD were attributable to the consumption of UPF in Brazil, corresponding to about 22% of the premature deaths from CVD and to 33% of the total premature all-cause deaths attributable to UPF intake among Brazilian adults. Reducing UPF consumption by 10% in the adult population would avert approximately 11% of the premature CVD deaths, equivalent to 2,100 deaths/year (95% UI, 697 to 4,511). A 20% reduction in UPF intake would avert approximately 21% of the premature CVD deaths or 4,100 deaths (95% UI, 1,413 to 8,047), and a 50% reduction in UPF intake would avert about 52% of the premature CVD deaths, corresponding to 9,900 deaths/year (95% UI, 3,682 to 17,820). If UPF consumption among adults was reduced to that of the first quintile of UPF intake in the baseline scenario, approximately 81% of the premature CVD deaths would be averted, corresponding to some 15,600 deaths/year (95% UI, 5,229 to 27,519).

**Conclusion:**

Our study estimated a high burden of premature CVD outcomes attributable to the consumption of UPF in Brazil. Our findings support food policies aimed at reducing the consumption of UPF, such as fiscal and regulatory policies, which are imperative to prevent CVD in Brazil.

## Introduction

As defined by the Nova food classification system, which considers the purpose and extent of industrial food processing, ultra-processed foods (UPF) are industrially manufactured formulations typically ready for consumption made of ingredients derived from foods (oils, fats, sugars, starch, protein isolates) and food additives with cosmetic function, containing little or no whole natural food (1). The ingredients and industrial techniques in their fabrication aim to create low-cost production, profitable, and extremely palatable and convenient products, which have gradually replaced unprocessed or minimally processed foods and culinary preparations in different countries (2).

Recent meta-analyses have consistently found significant dose-response associations between the dietary share of UPF and increased risk of all-cause deaths and non-communicable diseases, including cardiovascular diseases (CVD) (3–6). UPF are associated with an overall deterioration of nutritional quality of the diets (7) and a plethora of other postulated mechanisms associated with the presence of non-sugar sweeteners, emulsifiers and other additives (8), contaminants newly formed during processing or released from synthetic packaging (9, 10), and significant changes to the food matrix, leading to a highly degraded physical structure of food products (11). As a consequence, UPF are associated with low satiety potential, high glycemic loads (12), and inflammatory diseases, such as inflammatory bowel diseases and metabolic syndrome, possibly through modified gut microbiota and host–microbiota interactions (13). Specifically, regarding CVD risks, the mechanisms of action of UPF are not limited to the so-called critical nutrients, such as sodium, sugars and unhealthy fats, and include dysglycemia, insulin resistance, hypertension, and increased risk of obesity (14).

The Nova food classification has been increasingly used in dietary surveys and studies, including cohorts and meta-analyses in different countries, and, in Brazil, it was incorporated in the national dietary surveys (15, 16) and it provides the rationale for the National Dietary Guidelines (17, 18).

Over half of the total dietary energy consumed in certain high-income countries come from UPF, while sales of UPF have risen particularly in middle-income countries (19). In Brazil, the contribution of UPF to total energy intake increased by one third from 2002/2003 to 2017/2018, reaching 19.4% of the total energy (20, 21).

Despite the existing modeling studies to estimate the potential impact of specific dietary risk factors, especially of macro and micronutrient intakes and specific food groups (22), the health effects of dietary patterns based on the purpose and extent of food processing on morbimortality are scarce (23–25). Our previous study estimated that, approximately, 10.5% (57 thousand) of the all-cause premature deaths in 2019 were attributable to the consumption of UPF. Reducing the contribution of UPF to the total energy intake by 10 to 50% would, respectively, avert some 5.9 thousand to 29.3 thousand deaths (10.3% to 51.4% of the attributable deaths) in the year of reference (25). Alternatively, if UPF intake reached levels of consumption such as those from Mexico (29.8%) or the United States (57.0%), the attributable deaths would double or quadruple, respectively (26–28).

In this study, we aimed to estimate premature deaths, incidence, and disability adjusted life-years (DALYs) from CVD attributable to the consumption of UPF in Brazilian adults in 2019. We also estimated the potential impact of alternative scenarios of consumption of UPFs on CVD prevention.

## Materials and methods

### Study design

This study adapted a previously validated comparative risk assessment model (25) to estimate the burden of CVD deaths, new cases, and DALYs attributable to the consumption of UPF and to estimate the potential impact of reducing the consumption of UPF by 10, 20, and 50% and to the 1st quintile of UPF intake of the baseline scenario on these indicators.

The datasets used in the models are described in detail in Table 1, and the modeling details are presented in the Supplementary material. The modeling process involves (i) estimating the baseline intakes of UPF using dietary survey data representative of the Brazilian population, (ii) estimating the changes in UPF intake for each age- and sex-group for each counterfactual scenario; and finally (iii) estimating the effect of changes in UPF intake on the major CVD outcomes (ischaemic heart disease and stroke) through comparative risk assessment analysis.

**TABLE 1:** Comparative risk assessment model input parameters to estimate the burden of cardiovascular diseases attributable to the consumption of ultra-processed foods in Brazil, 2019.

Model inputs	RR	Source
**Baseline characteristics**		
Population count (by age and sex)		Brazilian Population Estimates (29)
Deaths, incident cases and DALYs (by age and sex)		Global Burden of Disease Study (GBD) (30)
Ultra-processed food intake (by age and sex)		Brazilian Dietary Survey 2017-2018 (16)
Ischaemic heart disease	1.29 (1.12-1.48)	Pagliai et al. (4)
Stroke	1.34 (1.07-1.68)	Pagliai et al. (4)

RR, relative risks; DALYs, disability adjusted life years.

### Consumption of ultra-processed foods

The consumption of foods and beverages in Brazilian were obtained through a 2 non-consecutive 24-hour food recall from the Brazilian Dietary Survey 2017–2018 for adults by sex and age-groups (30–34, 35–39, 40–44, 45–49, 50–54, 55–59, 60–64, and 65–69 years) (16). Foods and beverages were classified based on the NOVA classification into 4 major groups: unprocessed or minimally processed foods, processed culinary ingredients, processed foods, and UPF (1). The contribution of UPF to total energy intake was computed as the ratio of the mean energy from UPF food group over the mean total energy intake (Supplementary Table 1).

### Comparative risk assessment analysis

Within each sex-and-age-stratum, we calculated the estimated relative risks (RR) for UPF intake and coronary heart disease (CHD) and stroke considering intervals of 0.1% of participation of UPF in the diets from 0.0 (RR = 1.00 for CHD and stroke) to 22.0% (RR = 1.29 for CHD and RR = 1.34 for stroke), according to a meta-analysis by Pagliai et al. (4), and extrapolated the RR for contributions up to 100%. The meta-analysis was chosen because it provides robust risk factor-disease evidence through the comparison of well-defined “high risk” vs “low risk” groups. This meta-analysis was based on a random-effects model for pooled analysis of the association of ultra-processed food consumption with increased risk of deaths from ischaemic heart disease and stroke from three prospective cohort studies (31–33), including 2,501 cases.

The distribution of UPF consumption in each age- and sex-stratum considered a log-linear function for the mean participation of UPF in the energy of the diet and its standard deviation and the corresponding national population.

Finally, within each sex-and-age-stratum and for each scenario, we estimated the potential impact fraction (PIF) for the outcomes (o) in each age group (a) and sex (s) through the following formula:


$$P\,I\,F_{o\,a\,s}=\frac{\int_{x=0}^{m}R\,R_{o\,a}\,\left(x\right)\,P_{a\,s}\,\left(x\right)\,dx-\int_{x=0}^{m}R\,R_{o\,a}\,\left(x\right)\,P_{a\,s}^{\prime }\,\left(x\right)\,dx}{\int_{x=0}^{m}R\,R_{o\,a}\,\left(x\right)\,P_{a\,s}\,\left(x\right)\,dx}$$

Where: *Pas(x)* and *P’as(x)* are the UPF intake distributions at the baseline and in the counterfactual scenario. *RRoa(x)* is the RR as a function of UPF participation in the energy of the diet specific for outcome (*o*) and age.

The model used estimates and uncertainties of number of disease events (deaths, incidences, and DALYs) in Brazil during 2019 from the Global Burden of Disease Study (30) (Supplementary Tables 2–4). The averted number of new cases of CHD and stroke was computed by multiplying an age, sex, and cause-specific PIF by the baseline number of events for the same sex and age stratum (Supplementary Tables 2–4). The total numbers of new CVD events averted were calculated as the sum of estimates over all strata and we summed CHD and stroke estimates to generate estimates for total CVD.

The outcomes for individuals with less than 30 years of age were excluded because most CVD events occur among adults after this age threshold. Events among individuals over 70 years of age were also excluded to account only for the premature (preventable in principle) CVD deaths, incidence, and DALYs attributable to UPF intake (34).

The model also incorporated probabilistic sensitivity analyses using a Monte Carlo approach for estimating the uncertainty of different model parameters and population heterogeneity to be propagated to the outputs using the Ersatz program (*n* = 5,000) (35, 36). For each simulation, the simulation works through producing a draw from the distributions of (a) baseline participation of UPF intake in the energy of the diet, (b) prevalence and incidence of CHD and stroke in each stratum, (c) the RR of UPF intake and CHD and stroke outcomes, (d) the current number of events (deaths, incident cases, and DALYs) for each outcome. Each set of draws from the Monte Carlo analyses were incorporated in the estimated PIFs and averted events of each outcome for each age-sex stratum so results were reported for the median and the 95% uncertainty intervals (UI) and rounded to the nearest hundred.

Finally, the robustness of the model was assessed through deterministic sensitivity analyses, by changing key model assumptions and inputs. We evaluated the impact of higher minimum theoretical and higher and lower maximum UPF intake thresholds for the RR parametrization (10.0% ± 4.1% and 12.0% ± 5.0% or 20.0% ± 8.2%and 24.0% ± 10.6%, respectively). Lastly, we explored lower and higher RR for UPF intake and CVD outcomes (10% differences) than estimated in the primary model.

## Results

In 2019, a total of 88,438 Brazilian adults aged 30 to 69 years died prematurely from the major CVD (ischaemic heart disease and stroke). The contribution of ultra-processed foods to total energy intake of Brazilian adults decreases tended to decrease with age, for both men and women, and ranged from 13% to 21% of the total energy intake (Supplementary Table 3).

We estimated that approximately 19,200 premature deaths (95% UI, 7,097 to 32,353), 74,900 new cases (95% UI, 25,983 to 128,725), and 883,000 DALYs/year (95% UI, 324,279 to 1,492,593) from CVD were attributable to the consumption of UPF in Brazil in 2019 (Table 2), corresponding to, approximately, 22% of the premature CVD outcomes and one third of the deaths from all causes attributable to UPF.

**TABLE 2:** Estimated burden of ultra-processed foods on cardiovascular disease events among Brazilian adults from 30 to 69 years of age (deaths, incident cases and DALYs) in 2019.

Metric and disease		No. of events averted (95% UI)	
	
	Men	Women	Total
**Deaths/year**			
Total CVD	10,700 (4,735-16,752)	8,500 (2,361-15,601)	19,200 (7,097-32,353)
Ischaemic heart disease	6,900 (3,025-10,829)	4,500 (1,276-8,391)	11,400 (4,301-19,220)
Stroke	3,800 (1,710-5,923)	4,000 (1,085-7,209)	7,800 (2,795-13,133)
**Incident cases/year**			
Total CVD	31,900 (14,153-50,056)	43,000 (11,830-78,669)	74,900 (25,983-128,725)
Ischaemic heart disease	19,800 (8,724-31,232)	20,600 (5,757-38,087)	40,400 (14,481-69,319)
Stroke	12,100 (5,429-18,824)	22,300 (6,073-40,582)	34,400 (11,502-59,406)
**DALYs/year**			
Total CVD	479,300 (212,972-752,662)	403,700 (111,306-739,931)	883,000 (324,279-1,492,593)
Ischaemic heart disease	308,700 (136,439-486,890)	205,700 (57,498-380,410)	514,400 (193,937-867,300)
Stroke	170,600 (76,533-265,772)	198,000 (53,808-359,521)	368,600 (130,342-625,293)

CVD, cardiovascular disease; UI, uncertainty intervals; DALYs, disability adjusted life years.

Considering all premature deaths from CVD attributable to UPF, 59% were from ischaemic heart disease and 56% were among men. Approximately 54% of the premature incident cases of CVD attributable to UPF were from ischaemic heart disease and mostly among women (57%). Finally, 58% of the DALYs attributable to UPF intake were from ischaemic heart disease and, also, mostly among men (54%).

**FIGURE 1:**
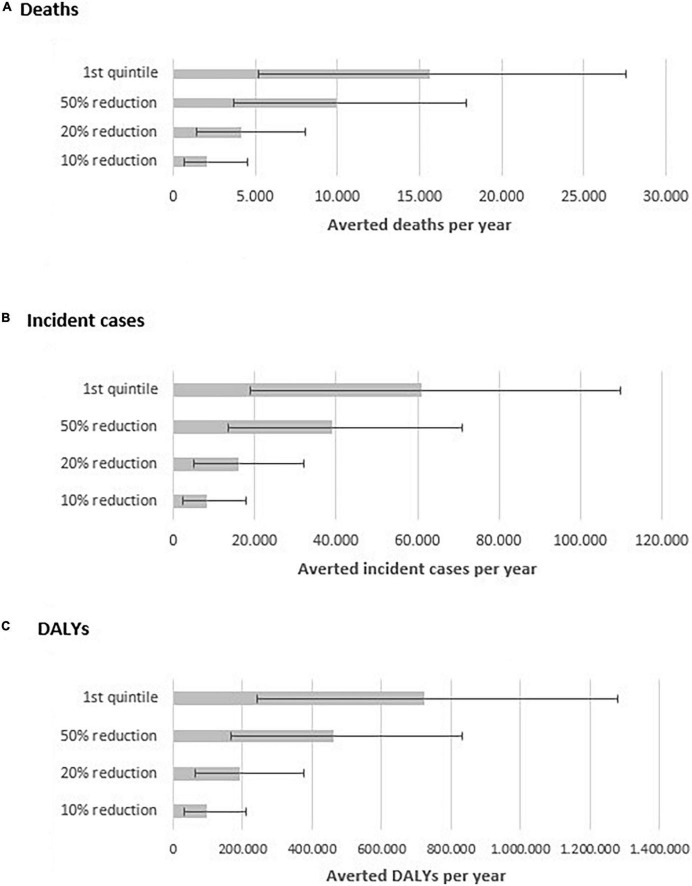
Estimated number of averted premature deaths **(A)** incident cases **(B)** and DALYs **(C)** per year by scenario of reduction of ultra-processed food intake in Brazil.

We estimated that a 10% reduction in the energy participation of UPF in the diet would avert 11% of all premature CVD events attributable to UPF (Figure 1), i.e., approximately, 2,100 deaths (95% UI, 697 to 4,511), 8,100 incident cases (95% UI, 1,413 to 8,047), and 96,400 DALYs (95% UI, 31,924 to 209,133) from CVD in 2019. A 20% in the energy participation of UPF would avert 21% of the premature CVD events attributable to UPF, i.e., approximately, 4,100 deaths (95% UI, 1,413 to 8,047), 16,200 incident cases (95% UI, 5,130 to 32,100), and 191,400 DALYs (95% UI, 65,127 to 374,968) from CVD in 2019. A 50% reduction in the energy participation of UPF in the diet is expected to prevent 52% of the premature CVD events attributable to UPF, corresponding to, approximately, 9,900 deaths (95% UI, 3,682 to 17,820), 38,900 incident cases (95% UI, 13,399 to 70,951), and 460,400 DALYs (95% UI, 168,345 to 830,583) from CVD in 2019. Finally, if the Brazilian population’s UPF consumption was reduced to that of the first quintile of the baseline scenario, some 15,600 premature deaths (95% UI, 5,229 to 27,519), 60,900 new cases (95% UI, 18,816 to 109,779), and 722,500 DALYs (95% UI, 239,993 to 1,279,187) from CVD would be averted in 2019.

### Sensitivity analyses

Considering the five different sensitivity analysis scenarios the modelled estimates of CVD events attributable to the consumption of UPF varied from -10.6% (10% higher RR) to +4.8% (12% higher minimum theoretical risk) compared to the primary model estimate. Other sensitivity analysis scenarios had relatively minor impact on the modeled estimates compared to the primary model (Supplementary Figure 1).

## Discussion

Based on this modeling study, the consumption of UPF is associated with a significant CVD burden in Brazil, contributing to about 22% of the premature CVD events or approximately 19,200 premature deaths, 74,900 incident cases and 883,000 DALYs in 2019. The premature CVD deaths attributable to UPF intake also represent 34% of the attributable all-cause deaths to UPF (25). Additionally, if UPF intake was progressively reduced by 10% up to 50%, we estimated that attributable CVD events would be reduced by 11 to 52%, and, if consumption was reduced to that of the first quintile of UPF distribution at the baseline (2017-2018), approximately 81% of the attributable CVD events would be averted.

Most previous modeling studies in Brazil and in other countries have estimated the CVD burden of specific-dietary factors associated with critical nutrients (37–41). Nevertheless, UPF intake is associated with disease outcomes, including CVD, independently of their low nutritional composition (excessive sodium, fat and sugar content) (42), so new modeling studies designed specifically to assess the impact of industrial food processing on health outcomes are needed. The association between the consumption of UPF and CVD has been evidenced by prospective cohort studies in different countries (32, 33, 43–45) and more recently in systematic reviews and meta-analyses (4–6). The effects of UPF on CVD and other cardiometabolic risk factors is likely mediated through biological mechanisms related to poor nutritional dietary quality, food additives, changes in the physical structure of foods, and other attributes of these foods that may affect health by altering serum lipid concentrations and causing inflammation, oxidative stress, dysglycemia, insulin resistance, and hypertension, among other outcomes (42). Therefore, this study has estimated the association between dietary patterns and CVD events, by incorporating the potential impacts of nutrients, food additives and industrial food processing on cardiovascular health that are not captured by other models (46).

In Brazil, as in many other low and middle-income countries, traditional fresh and minimally processed foods have been replaced by ready-to-(h)eat UPF over the last two decades (16, 47). Although there are few studies on the estimated impact of these dietary changes, our previous study estimated that if UPF intake increase by around 50% (to intakes similar to those of Mexico), the all-cause attributable deaths would almost double, while if UPF intake tripled (to the intakes equivalent to those in the United States), the attributable deaths would be increased by 250% (28). Also, other study has estimated that the impact of different reduction scenarios for saturated and trans fats, salt and added sugar from culinary ingredients, processed and ultra-processed foods could avert from 37.6 to 196.4 thousand deaths from CVD in Brazil, in 2,048 (24).

UPF intake is an important dietary risk factor that must be addressed through individual and populational preventive strategies such as changing food environments, strengthening the implementation of food-based dietary guidelines, and improving consumer knowledge, attitudes, and behaviour. Individual-level dietary strategies to change behavioral risk factors are very limited. Therefore, it is key for public policies to promote healthy food environments to reduce the intake of ultra-processed foods, considering the need to incentive the consumption of fresh and minimally processed foods and to discourage UPF intake, through fiscal and regulatory policies. These policies may include the regulation of food publicity, the regulation of sales of unhealthy foods in school and work environments, the implementation of front of package nutritional labeling, subsidies to the production and commercialization of fresh local foods, and through the taxation of UPF (48–51). For example, in Chile, the purchase of foods high in calories declined by 23.8% after the implementation of the front of package nutritional warnings (52). In Mexico, sugary beverage consumption was reduced by 6.3% after a 10% tax to sugar-sweetened beverages (53).

Particularly in the national context, the Dietary Guidelines for the Brazilian Population play an important role in nutritional public policies, by recommending diets based on natural or minimally processed and avoiding the consumption of UPF (17). These recommendations must be implemented considering both individual and populational strategies and must guide health sector and intersectoral policies for healthy diet promotion. After Brazil, several countries and international organizations have adopted dietary guidelines based on the extension and purpose of food processing (54–56).

### Strengths and limitations

Comparative risk assessment models are well acknowledged ex ante evaluation tools for estimating the burden of dietary risk factors an to assess potential food policy implementation scenarios, that have been validated in literature and adapted to different country contexts (38, 57–59). Based on these methods, recent robust meta-analysis studies have provided estimates of the RR of UPF intake and several health outcomes, allowing the development and validation of the first modeling studies to assess the impact of dietary patterns based on the extent and purpose of food processing (25).

Additionally, the modeled data inputs nationally representative (demographic and food consumption data) and based on deaths, incident cases and DALYs were obtained from validated estimates from the GBD Study for Brazil (30). In particular, the UPF intake data was obtained from a nationally representative sample, based in two non-consecutive 24-h food recalls with strong quality control protocols (16). Finally, the RR used in the model were obtained from the recent meta-analysis based on cohort studies in various countries (4).

There are several limitations that should be considered when interpreting our results. First, we assumed the portability of the pooled RR, which were based on cohort studies from other countries, to estimate the PIF for Brazil (60). Second, we can not exclude the possibility of residual confounding in these RR estimates. In order to overcome part of these limitations, we incorporated the uncertainties of the RR estimates and of other data inputs in the model through Monte Carlo simulations (61). Third, comparative risk assessment models, when compared to dynamic modeling approaches, do not incorporate a timeframe properly, so they are not intended to estimate the projected future impacts of changes in the risk factors and do not consider the possible time lag between changes in risk exposure and disease outcomes. Finally, comparative risk assessment models do not account for recurring events and do not consider the influence of interactions between individuals, populations or their environments and the potentials health equity impacts. This model allows a comparable and consistent estimation of premature CVD events attributable to the consumption of UPF that can be applied to different contextsto estimate the population health impact of changes in the diets. Therefore, the model represents a helpful tool for researchers and policymakers to understand the impact of dietary patterns on health outcomes and to develop and assess context-specific prevention policies. Of note, our study was concentrated solely on estimating the impact of UPF intake on CVD outcomes. Future modeling studies must also include other disease outcomes associated to UPF, such as obesity, diabetes, and cancers, in order to better estimate the overall health burden of industrial food processing and support policies for improving the food environment.

## Conclusion

UPF intake is associated with an important CVD burden in Brazil. We estimated that, approximately, one third of the CVD events per year are attributable to the consumption of UPF in the country. This study provides evidence regarding the overall impact of industrial food processing on preventable CVD outcomes, supporting the Brazilian Dietary Guidelines, especially by avoiding the consumption of UPF. Our findings suggest that reducing UPF consumption should be a food policy priority within the strategies for improving cardiovascular health, achieving population health gains, and reducing preventable CVD events in Brazil.

## Data availability statement

The original contributions presented in this study are included in the article/Supplementary material, further inquiries can be directed to the corresponding author.

## Author contributions

EN, ML, and LR conceived the idea and contributed to the design of the work. EN developed and validated the comparative risk assessment model and drafted the first draft of the manuscript. EN and ML contributed to the acquisition, analysis, and interpretation of data for the work. All authors have reviewed the draft manuscript and approved the final document.
