# Supplementary material for: The estimated burden of ultra-processed foods on cardiovascular disease outcomes in Brazil: A modeling study
Source: Front Nutr. 2022 Nov 17;9:1043620. doi: 10.3389/fnut.2022.1043620 (PMC9712187; doi:10.3389/fnut.2022.1043620)
Supplement: Supplementary file 1 [file Data_Sheet_1.docx]

**Supplementary Materials**

**The estimated burden of ultra-processed foods on cardiovascular disease outcomes in Brazil: A modeling study**

**Eduardo Nilson, Gerson Ferrari, Maria Laura da Costa Louzada, Renata Bertazzi Levy, Carlos Augusto Monteiro, Leandro F.M. Rezende**

**Text A. Data sources and modelling steps**

**Baseline food consumption**

The food recall data for Brazilian adults was collected by the National Household Budget Survey (POF, Pesquisa de Orçamentos Familiares), carried out by the Brazilian Institute of Geography and Statistics (IBGE), between July 2017 and July 2018 using a probabilistic sample of 20,112 households (46,164 individuals), were used as the basis of this study (1). The food recall consisted of interviews in two non-consecutive days for all individuals in the sample, registering all foods consumed and their quantities.

The POF 2017-2018 used the same complex cluster sampling of the POF 2008-2009 survey, including adjustments to the Master Sample of Household Surveys (or Common Sample) and to the aggregated sectors of IBGE household surveys. This Common Sample is a set of primary sampling units), with the aim of stratifying the households with geographic and socio-economic homogeneity based on data from the 2000 Demographic Census.

The POF 2017-2018 sample was set as a subsample of the Master Sample, yet the sampling system maintained the comparability to the stratification of previous surveys, such as POF 2002-2003 and 2007-2008. The subsample of primary sampling units (PSU) for 2017-2018 was selected by simple random sampling in each stratum. In the adopted sampling plan, permanent private households were considered as secondary sampling units, which were also selected through simple random sampling without replacement for each selected PSU.

The number of sectors or tracts drawn for each stratum were proportional to the total number of households in the strata, with the provision of keeping at least three tracts in the sample of each stratum. Households in each tract were drawn by simple random sampling, without replacement. The number of households interviewed, per sector, was fixed according to the area of the research (12 households in the urban tracts, 16 in the rural tracts). Also, PSUs are distributed throughout the four quarters (three-month-periods) of the study, reproducing seasonal variations in income and spending on food (and other products) in each stratum and ensuring that all geographic and socio-economic strata are represented. Therefore, the POF estimates refer to aggregates of households representing regions, states or even municipalities (such as state capitals). It was decided to use aggregates of households as the unit of the study, corresponding to the households studied in each of the 550 sample strata of the study.

The data on the foodstuffs were divided into different groups in the POF 2017-2018 (2). Based on this division and after being converted into energy, the records were reunited in the food groups according to the NOVA food classification: *in natura* and minimally processed foods, processed culinary ingredients, processed foods and ultra-processed foods (Supplementary Table S1)(3).

**Counterfactual scenarios of food consumption**

Counterfactual (alternative scenarios) of ultra-processed food consumption were estimated by:

- Percentage reductions were estimated reducing the respective contribution of ultra-processed foods intake from baseline and estimating the standard deviation of the new scenario as proportional to the standard deviation of the baseline to its respective mean value.
- Reduction to the consumption of the first quintile was estimated by limiting the maximum ultra-processed food consumption to the consumption at the upper limit of the first quintile of baseline distribution estimating the standard deviation of the distribution considering that the new upper limit of consumption corresponds to the average ultra-processed food intake plus three standard deviations of the average (Supplementary Table S2).

**Interpretation of counterfactual scenarios**

Although changes in UPF intake may seem to be linear in some ranges of the distribution of UPF consumption, the potential impact factors (PIF), which depends on the prevalence of the risk factor and its relative risk, does not change linearly because of the changes in the relative risks for each new point of the intake distribution. For example, in the upper side of the distribution of UPF intake, the scenarios far from linear: higher UPF participation in the total energy of the diet represent larger impacts on attributable deaths (a 50% increase in UPF intake would double the attributable deaths and a 2.5-time increase would almost quadruple the attributable deaths). (4)

The distribution of RR is exponential with a small curvature, so that in certain ranges, it may appear to be close to linear. Nevertheless, the new distributions for the scenarios of changes in UPF intake will fall into different relative risks for CVD mortality and the resulting changes in PIFs and in attributable CVD outcomes will not be linear.

**Supplementary Table S1 - Contribution of food groups and subgroups to the total energy intake according to the NOVA classification** **– 2017-2018**

|  | **Contribution to the total energy intake (%)** | | |
| --- | --- | --- | --- |
| **Food groups and subgroups** | **Total** | **Men** | **Women** |
| **Fresh and minimally processed foods** | **53.4** | **54.1** | **52.8** |
| Rice | 11.1 | 11.9 | 10.4 |
| Beef | 7.4 | 7.7 | 7.1 |
| Beans | 6.6 | 7.3 | 6.0 |
| Poultry | 5.4 | 5.5 | 5.4 |
| Fruits | 3.1 | 2.4 | 3.8 |
| Pasta | 2.8 | 2.4 | 3.1 |
| Milk | 2.5 | 2.5 | 2.4 |
| Vegetables | 1.9 | 1.7 | 2.0 |
| Pork | 1.8 | 2.0 | 1.6 |
| Roots and tubers | 1.8 | 1.6 | 1.9 |
| Fruit juice (100% fruit content) | 1.6 | 1.5 | 1.7 |
| Eggs | 1.4 | 1.5 | 1.4 |
| Cassava flour | 1.4 | 1.6 | 1.2 |
| Fish | 1.1 | 1.0 | 1.1 |
| Corn, oat and other cereals | 1.1 | 1.1 | 1.1 |
| Wheat flour | 0.7 | 0.7 | 0.7 |
| Coffee and tea | 0.5 | 0.4 | 0.6 |
| Other flours | 0.4 | 0.3 | 0.4 |
| Edible viscera | 0.3 | 0.3 | 0.3 |
| Lentils, chicken pea and other | 0.2 | 0.2 | 0.2 |
| Nuts and seeds | 0.2 | 0.1 | 0.2 |
| Others | 0.3 | 0.2 | 0.3 |
| **Processed culinary ingredients** | **15.6** | **15.0** | **16.2** |
| Vegetable oil | 7.7 | 7.7 | 7.7 |
| Sugar | 5.8 | 5.4 | 6.1 |
| Butter | 1.0 | 0.9 | 1.0 |
| Fat | 0.3 | 0.4 | 0.3 |
| Others | 0.8 | 0.5 | 1.0 |
| **Processed foods** | **11.3** | **11.8** | **10.8** |
| French bread | 8.2 | 8.5 | 7.8 |
| Cheeses | 1.6 | 1.5 | 1.7 |
| Beer and wine | 0.7 | 0.9 | 0.4 |
| Processed meat (salted, smoked, dried) | 0.4 | 0.5 | 0.4 |
| Canned fruits and vegetables | 0.2 | 0.2 | 0.3 |
| Others | 0.2 | 0.2 | 0.2 |
| **Ultra-processed foods** | **19.7** | **19.1** | **20.3** |
| Margarine | 2.1 | 2.0 | 2.3 |
| Salted crackers | 1.7 | 1.8 | 1.7 |
| Breads | 1.6 | 1.7 | 1.6 |
| Cookies | 1.4 | 1.2 | 1.6 |
| Hams | 1.3 | 1.5 | 1.1 |
| Confectionary deserts | 1.1 | 1.2 | 1.0 |
| Soft drinks | 1.1 | 0.9 | 1.2 |
| Hotdogs, hamburgers and sandwiches | 0.9 | 1.0 | 0.9 |
| Sweetened dairy drinks | 0.7 | 0.7 | 0.7 |
| Pizza | 0.6 | 0.6 | 0.6 |
| Fried and baked snacks | 0.6 | 0.5 | 0.7 |
| Other sugar sweetened beverages | 0.4 | 0.4 | 0.4 |
| Ready or semi-ready meals | 0.4 | 0.3 | 0.5 |
| Sauces | 0.4 | 0.4 | 0.4 |
| Cakes and pies | 2.1 | 2.0 | 2.3 |
| Others | 1.7 | 1.8 | 1.7 |

**Supplementary Table S2. Counterfactual scenarios for changes in the contribution of ultra-processed foods to total energy intake in Brazilian adults aged 30 to 69 (2017-2018).**

|  | **10% reduction** | | **20% reduction** | | **50% reduction** | | **1st Quintile** | |
| --- | --- | --- | --- | --- | --- | --- | --- | --- |
|  | **UPF intake (%)** | **SD UPF** | **UPF intake (%)** | **SD UPF** | **UPF intake (%)** | **SD UPF** | **UPF intake (%)** | **SD UPF** |
| **Men** |  |  |  |  |  |  |  |  |
| **15-19y** | 22.59 | 9.97 | 17.57 | 7.75 | 12.55 | 5.54 | 4.68 | 2.06 |
| **20-24y** | 20.54 | 9.07 | 15.97 | 7.06 | 11.41 | 5.04 | 4.24 | 1.87 |
| **25-29y** | 20.10 | 8.63 | 15.63 | 6.71 | 11.16 | 4.79 | 4.23 | 1.82 |
| **30-34y** | 16.58 | 6.77 | 12.89 | 5.27 | 9.21 | 3.76 | 3.58 | 1.46 |
| **35-39y** | 16.71 | 6.74 | 13.00 | 5.24 | 9.28 | 3.75 | 3.64 | 1.47 |
| **40-44y** | 13.95 | 5.70 | 10.85 | 4.43 | 7.75 | 3.16 | 3.02 | 1.23 |
| **45-49y** | 16.24 | 6.40 | 12.63 | 4.98 | 9.02 | 3.55 | 3.57 | 1.40 |
| **50-54y** | 13.90 | 5.76 | 10.81 | 4.48 | 7.72 | 3.20 | 2.99 | 1.24 |
| **55-59y** | 13.19 | 5.42 | 10.26 | 4.21 | 7.33 | 3.01 | 2.84 | 1.17 |
| **60-64y** | 11.67 | 5.09 | 9.08 | 3.96 | 6.48 | 2.83 | 2.42 | 1.05 |
| **65-69y** | 12.80 | 5.13 | 9.95 | 3.99 | 7.11 | 2.85 | 2.79 | 1.12 |
| **70-74y** | 12.96 | 5.23 | 10.08 | 4.07 | 7.20 | 2.91 | 2.82 | 1.14 |
| **75-79y** | 11.75 | 4.49 | 9.14 | 3.49 | 6.53 | 2.49 | 2.63 | 1.00 |
| **80y+** | 11.47 | 5.17 | 8.92 | 4.02 | 6.37 | 2.87 | 2.32 | 1.04 |
|  |  |  |  |  |  |  |  |  |
| **Women** |  |  |  |  |  |  |  |  |
| **15-19y** | 23.60 | 10.05 | 18.35 | 7.82 | 13.11 | 5.58 | 4.99 | 2.13 |
| **20-24y** | 22.50 | 9.27 | 17.50 | 7.21 | 12.50 | 5.15 | 4.84 | 1.99 |
| **25-29y** | 19.81 | 8.58 | 15.40 | 6.68 | 11.00 | 4.77 | 4.14 | 1.79 |
| **30-34y** | 18.87 | 7.83 | 14.67 | 6.09 | 10.48 | 4.35 | 4.04 | 1.68 |
| **35-39y** | 17.07 | 6.88 | 13.28 | 5.35 | 9.48 | 3.82 | 3.70 | 1.49 |
| **40-44y** | 16.67 | 7.00 | 12.97 | 5.44 | 9.26 | 3.89 | 3.54 | 1.49 |
| **45-49y** | 16.57 | 6.70 | 12.88 | 5.21 | 9.20 | 3.72 | 3.58 | 1.45 |
| **50-54y** | 15.62 | 6.32 | 12.15 | 4.91 | 8.68 | 3.51 | 3.38 | 1.37 |
| **55-59y** | 14.62 | 5.64 | 11.37 | 4.38 | 8.12 | 3.13 | 3.25 | 1.25 |
| **60-64y** | 14.66 | 5.94 | 11.40 | 4.62 | 8.14 | 3.30 | 3.17 | 1.29 |
| **65-69y** | 14.40 | 5.79 | 11.20 | 4.50 | 8.00 | 3.21 | 3.15 | 1.26 |
| **70-74y** | 14.58 | 5.94 | 11.34 | 4.62 | 8.10 | 3.30 | 3.14 | 1.28 |
| **75-79y** | 13.58 | 5.66 | 10.56 | 4.40 | 7.54 | 3.14 | 2.90 | 1.21 |
| **80y+** | 16.07 | 6.44 | 12.50 | 5.01 | 8.93 | 3.58 | 3.50 | 1.40 |

.

**Text B**

**Comparative risk assessment analysis**

The comparative risk assessment model was developed and validated for estimating the impact of ultra-processed food consumption on all-cause mortality in Brazil. The model can be adapted to different countries using similar inputs to specific realities (population, mortality and food consumption). By addressing all-cause mortality, the model is intended to estimate the total burden of ultra-processed foods and support research on food policies intended to reduce ultra-processed food consumption and compare different scenarios of changes in diet.

The first step of the modeling is to estimate the estimated relative risks for all-cause mortality within each sex-and-age-stratum assuming that the equivalent to the theoretical minimum risk exposure level for ultra-processed foods consumption would be zero and considering discrete intervals of 0.1% of contribution of ultra-processed foods to total energy intake from 0.0 (RR=1.00 for CHD and stroke) to 22.0% (RR=1.29 for CHD and RR= 1.34 for stroke), based on the summary relative risk from a recent meta-analysis (5) and the available distribution of the contribution of ultra-processed foods to total energy intake (6), and extrapolated the RR for contributions up to 100%.

The distribution of the relative risks was considered log-linear for the interval so that the increase in the contribution of ultra-processed foods to total energy intake corresponded an equivalent increase in the RR for all-cause mortality, based on an increase in the natural logarithm of the relative risk of outcome in age a per 0.1% increase in the consumption of ultra-processed foods (Table S) through the formula:

$${RR}_{oas}= {RR}_{ma}^ (i/r)$$

Where RR_oas_ is the estimated relative risk for the interval of the UPF intake distributions, RR_ma_ is the relative risk from the meta-analysis, (i) is the interval point and (r) is the range of the RR_ma_ distribution.

Then, similarly to other validated macrosimulation models that address dietary risk factors (7), we modelled the distribution of UPF consumption using a log-linear function for the mean contribution of ultra-processed foods to total energy intake of the diet and its standard deviation and the national population (Table S2).

Based on these intermediate outputs of the model, we calculated the estimated potential impact fraction (PIF) for mortality outcome (o) in each age group (a) and sex (s) stratum for each counterfactual scenario through the following formula:

$${PIF}_{oas}= \frac{\int_{x=0}^{m} {RR}_{oa}\left( x \right)P_{as}\left( x \right)dx-\int_{x=0}^{m} {RR}_{oa}\left( x \right)P_{as}^{'}\left( x \right)dx}{\int_{x=0}^{m} {RR}_{oa}\left( x \right)P_{as}\left( x \right)dx}$$

Where: Pas(x) and P’as(x) are the UPF intake distributions at the baseline and in the counterfactual scenario, respectively. RRoa(x) is the relative risk for all-cause mortality (outcome o) as a function of UPF participation in the energy of the diet, according to age group.

We utilized estimates of the total number of deaths, incident cases and DALYs in Brazil during 2019 from the Global Burden of Disease Study (8) (Supplementary Tables S3-S5). The averted CVD events in each counterfactual scenario were computed by multiplying an age- and sex-specific PAF by the baseline total number of deaths for the same stratum. For each scenario, the model generated the total numbers of deaths prevented or postponed as outputs. All modelling input parameters are detailed in Supplementary Table S6 and the main model assumptions are stated in Supplementary Table S7. Additionally, Supplementary Tables S8-S10 summarize key intermediate estimates of the model and Supplementary Tables S11-S14 detail the estimates of the attributable burden of UPF on CVD and the averted deaths from each UPF reduction scenario according to age and sex groups.

As modeled estimations of the impact of ultra-processed food consumption, the results were presented for Brazilian adults aged 30 to 79 years for 2019, rounded to 2 significant digits (uncertainty intervals, UI 95%, were not rounded). The outcomes for individuals with less than 30 years of age were excluded from the analysis because of the onset of most cardiovascular events, and disease outcomes and excluded the individuals over 70 years of age to account only for the premature deaths attributable to UPF intake.

Considering the uncertainty of outcomes in the model, probabilistic sensitivity analysis is recommended in order to explore the potential effects of reducing ultra-processed food consumption on all-cause mortality. Probabilistic sensitivity analysis requires a stochastic (random) variation of parameters based on the sizes of the effects obtained from the literature, as using Monte Carlo simulations. By using this methodology, the model results are recalculated iteratively and uncertainty intervals of 95% (UI 95%) are generated for the median using the bootstrap percentile method.

The parameter uncertainty in all modelled estimates was quantified using Monte Carlo iterations with the Ersatz program (n = 5,000). For each simulation, the simulation works thorough producing a draw from the distributions of a) baseline contribution of ultra-processed foods to total energy intake of the diet, b) the relative risks of UPF intake and CVD mortality, c) the current number of deaths. Each set of draws were used to calculate PIF and averted events of each outcome for each age-sex stratum and results were reported for the 50th, 2.5th and 97.5th percentiles of estimates across all simulations as the central estimate and 95% uncertainty intervals (UI), respectively.

Finally, the robustness of the model was assessed through deterministic sensitivity analyses, by changing key model assumptions and inputs. We evaluated the impact of higher minimum and lower maximum theoretical risks for UPF intake and CVD outcomes (12.0% and 20.0%, respectively). Lastly, we explored lower and higher RR for UPF intake and CVD outcomes (10% differences) than estimated in the primary model. (Supplementary Figure 1). (9)

Finally, we analyzed the model development and its inputs using the STREAMS-P tool, that provides guidelines on a standardized approach for assessing and reporting how methodological decisions that might affect the validity of the estimates. The information of the analysis is summarized in Supplementary Table 15.

**Supplementary Table S3. Total population (2019), cardiovascular disease (CVD) mortality (2019) and contribution of ultra-processed foods to total energy intake in Brazilian adults aged 30 to 69 (2017-2018).**

|  |  |  | | **Baseline** | |  | |  |
| --- | --- | --- | --- | --- | --- | --- | --- | --- |
|  | **Population** | **CVD mortality** | | **UPF intake (%)** | **SD UPF** | **CI 95%** | |  |
| **Men** | | |  | | | | | |
| **15-19y** | 8,710,079 | 157 | | 25.10 | 11.07 | 23.31 | 26.90 |  |
| **20-24y** | 8,621,967 | 305 | | 22.82 | 10.08 | 20.75 | 24.89 |  |
| **25-29y** | 8,634,062 | 440 | | 22.33 | 9.59 | 20.80 | 23.86 |  |
| **30-34y** | 8,816,350 | 800 | | 18.42 | 7.53 | 17.22 | 19.61 |  |
| **35-39y** | 7,879,629 | 1,508 | | 18.57 | 7.49 | 16.70 | 20.43 |  |
| **40-44y** | 6,882,229 | 2,714 | | 15.50 | 6.33 | 14.38 | 16.63 |  |
| **45-49y** | 6,266,088 | 4,338 | | 18.05 | 7.11 | 16.44 | 19.65 |  |
| **50-54y** | 5,659,602 | 7,312 | | 15.44 | 6.40 | 14.32 | 16.57 |  |
| **55-59y** | 4,678,702 | 10,223 | | 14.65 | 6.02 | 13.57 | 15.73 |  |
| **60-64y** | 3,655,025 | 13,561 | | 12.97 | 5.65 | 11.86 | 14.07 |  |
| **65-69y** | 2,672,043 | 15,488 | | 14.22 | 5.70 | 12.96 | 15.48 |  |
| **70-74y** | 1,793,543 | 16,078 | | 14.40 | 5.81 | 12.62 | 16.19 |  |
| **75-79y** | 1,222,286 | 16,044 | | 13.06 | 4.99 | 11.37 | 14.75 |  |
| **80y+** | 1,246,775 | 31,301 | | 12.74 | 5.74 | 10.52 | 14.97 |  |
|  |  |  | |  |  |  |  |  |
| **Women** | | |  | | | | | |
| **15-19y** | 8,430,049 | 88 | | 26.22 | 11.17 | 24.45 | 27.99 |  |
| **20-24y** | 8,434,408 | 176 | | 25.00 | 10.30 | 23.46 | 26.54 |  |
| **25-29y** | 8,542,758 | 261 | | 22.01 | 9.54 | 20.68 | 23.33 |  |
| **30-34y** | 8,821,067 | 449 | | 20.96 | 8.71 | 19.26 | 22.66 |  |
| **35-39y** | 7,976,620 | 907 | | 18.97 | 7.65 | 17.87 | 20.06 |  |
| **40-44y** | 7,062,001 | 1,615 | | 18.53 | 7.78 | 17.33 | 19.72 |  |
| **45-49y** | 6,536,311 | 2,677 | | 18.41 | 7.44 | 17.04 | 19.78 |  |
| **50-54y** | 6,027,703 | 3,916 | | 17.36 | 7.02 | 15.93 | 18.79 |  |
| **55-59y** | 5,120,890 | 5,487 | | 16.24 | 6.26 | 15.18 | 17.29 |  |
| **60-64y** | 4,141,984 | 7,697 | | 16.29 | 6.60 | 15.17 | 17.41 |  |
| **65-69y** | 3,172,657 | 9,746 | | 16.00 | 6.43 | 14.51 | 17.49 |  |
| **70-74y** | 2,283,021 | 11,338 | | 16.20 | 6.59 | 14.69 | 17.72 |  |
| **75-79y** | 1,691,282 | 13,467 | | 15.08 | 6.29 | 13.44 | 16.73 |  |
| **80y+** | 2,062,198 | 40,165 | | 17.86 | 7.15 | 13.66 | 22.07 |  |

**Supplementary Table S4. DALYs from coronary heart disease (CHD) and stroke in Brazil (GBD, 2019).**

|  | **DALYs (UI 95%)** | |
| --- | --- | --- |
|  | **CHD** | **Stroke** |
| **Men** |  |  |
| **15-19y** | 7,339 (6,499 to 8,222) | 8,999 (7,830 to 10,253) |
| **20-24y** | 13,970 (12,609 to 15,497) | 12,482 (10,901 to 14,156) |
| **25-29y** | 20,638 (18,903 to 22,591) | 15,940 (14,126 to 17,857) |
| **30-34y** | 40,423 (37,457 to 43,813) | 24,871 (22,182 to 27,867) |
| **35-39y** | 70,571 (65,588 to 76,293) | 41,398 (37,184 to 45,951) |
| **40-44y** | 113,594 (106,180 to 121,396) | 63,797 (58,174 to 70,026) |
| **45-49y** | 171,458 (161,046 to 182,757) | 87,887 (80,112 to 96,484) |
| **50-54y** | 249,072 (234,646 to 265,298) | 126,869 (116,425 to 138,361) |
| **55-59y** | 308,064 (288,766 to 328,876) | 159,586 (147,844 to 172,215) |
| **60-64y** | 325,826 (306,137 to 348,161) | 187,002 (172,842 to 201,101) |
| **65-69y** | 305,402 (286,542 to 325,267) | 195,404 (180,147 to 210,022) |
| **70-74y** | 257,571 (240,156 to 273,973) | 190,462 (175,416 to 204,882) |
| **75-79y** | 188,193 (172,301 to 201,781) | 159,133 (145,765 to 170,567) |
| **80y+** | 233,610 (200,138 to 255,596) | 207,366 (178,331 to 225,751) |
|  |  |  |
| **Women** |  |  |
| **15-19y** | 2,523 (2,255 to 2,799) | 8,484 (7,374 to 9,685) |
| **20-24y** | 4,887 (4,273 to 5,505) | 12,261 (10,636 to 14,203) |
| **25-29y** | 6,784 (6,073 to 7,524) | 16,133 (14,200 to 18,170) |
| **30-34y** | 14,324 (12,943 to 15,608) | 26,609 (23,920 to 29,657) |
| **35-39y** | 27,835 (25,412 to 30,223) | 45,619 (41,349 to 50,151) |
| **40-44y** | 48,109 (44,205 to 52,753) | 67,598 (61,918 to 72,813) |
| **45-49y** | 77,626 (71,356 to 84,055) | 84,097 (77,410 to 91,135) |
| **50-54y** | 107,048 (98,925 to 114,931) | 105,822 (96,420 to 115,388) |
| **55-59y** | 140,073 (129,761 to 151,225) | 122,959 (113,480 to 132,493) |
| **60-64y** | 174,779 (160,998 to 188,861) | 136,194 (125,506 to 147,017) |
| **65-69y** | 185,333 (170,924 to 198,991) | 145,806 (133,660 to 157,819) |
| **70-74y** | 179,442 (162,327 to 193,895) | 153,725 (139,689 to 165,979) |
| **75-79y** | 157,481 (140,922 to 171,171) | 151,628 (135,919 to 164,379) |
| **80y+** | 289,046 (235,854 to 321,269) | 283,259 (235,168 to 313,439) |

**Supplementary Table S5. Incident cases from coronary heart disease (CHD) and stroke in Brazil (GBD, 2019).**

|  | **Cases (UI 95%)** | |
| --- | --- | --- |
|  | **CHD** | **Stroke** |
| **Men** |  |  |
| **15-19y** | 351 (75 to 764) | 1,109 (702 to 1,639) |
| **20-24y** | 1,187 (733 to 1,835) | 1,327 (904 to 1,930) |
| **25-29y** | 2,010 (1,211 to 2,934) | 1,735 (1,140 to 2,640) |
| **30-34y** | 2,625 (1,761 to 3,528) | 2,803 (2,111 to 3,711) |
| **35-39y** | 2,922 (1,837 to 4,416) | 4,420 (3,215 to 5,873) |
| **40-44y** | 4,971 (3,723 to 6,399) | 6,561 (5,299 to 8,105) |
| **45-49y** | 8,204 (5,793 to 10,817) | 8,968 (6,781 to 11,773) |
| **50-54y** | 14,311 (11,455 to 17,633) | 12,514 (10,168 to 15,438) |
| **55-59y** | 21,223 (15,297 to 28,294) | 15,747 (11,929 to 20,513) |
| **60-64y** | 24,461 (19,841 to 30,202) | 18,019 (14,206 to 22,461) |
| **65-69y** | 24,429 (19,101 to 30,161) | 18,914 (13,419 to 25,615) |
| **70-74y** | 20,562 (16,564 to 24,582) | 17,079 (13,041 to 22,309) |
| **75-79y** | 14,686 (11,213 to 18,873) | 13,241 (9,698 to 17,340) |
| **80y+** | 17,331 (14,274 to 20,889) | 18,783 (14,719 to 23,153) |
|  |  |  |
| **Women** |  |  |
| **15-19y** | 278 (67 to 626) | 1,452 (937 to 2,097) |
| **20-24y** | 833 (445 to 1,364) | 1,771 (1,227 to 2,526) |
| **25-29y** | 1307 (761 to 2,009) | 2,327 (1,593 to 3,350) |
| **30-34y** | 1526 (947 to 2,139) | 3,703 (2,834 to 4,803) |
| **35-39y** | 1400 (785 to 2,309) | 5,741 (4,328 to 7,445) |
| **40-44y** | 2313 (1,597 to 3,152) | 7,683 (6,249 to 9,346) |
| **45-49y** | 3979 (2,755 to 5,310) | 9,484 (7,424 to 12,202) |
| **50-54y** | 7030 (5,515 to 8,877) | 11,823 (9,632 to 14,506) |
| **55-59y** | 10592 (7,395 to 14,314) | 13,544 (10,538 to 16,978) |
| **60-64y** | 13385 (10,767 to 16,646) | 14,844 (11,893 to 18,547) |
| **65-69y** | 14826 (11,131 to 18,658) | 15,281 (11,234 to 20,103) |
| **70-74y** | 13976 (11,209 to 16,806) | 14,886 (11,644 to 18,941) |
| **75-79y** | 11625 (8,842 to 15,341) | 13,420 (9,809 to 17,884) |
| **80y+** | 18320 (15,268 to 21,992) | 28,317 (22,383 to 34,839) |

**Supplementary Table S6**. Modelling input parameters

| **Model inputs** | **Relative risk** | **Source** |
| --- | --- | --- |
| Baseline characteristics |  |  |
| Demographics |  | Brazilian Population Estimates (IBGE) (10) |
| Deaths, incident cases and DALYs |  | Global Burden of Disease (GBD) (9) |
| Consumption of ultra-processed foods |  | National Household Budget Survey – Personal Food Intake - POF 2017-2018 (1) |
| Relative risks of all-cause mortality | CHD: RR=1.29 (1.12-1.48)  Stroke: RR= 1.34 (1.07-1.68) | (5) |

**Supplementary Table S7.** **Key assumptions and restrictions of the model that estimates the impact of ultra-processed food consumption on all-cause mortality.**

| **Category** | **Assumption/Restriction** | **Motivation** |
| --- | --- | --- |
| **Ultra-processed food consumption** | Log-linear distribution of consumption by age and sex groups. | Prior literature for dietary risk factor models (7)(9). |
|  | Ultra-processed food consumption has not significantly altered from 2017-2018 to 2019 | Lack of a detailed time series to estimate projections in the change in the consumption of NOVA food groups |
| **Counterfactual scenario design** | The relative proportion of the standard deviations to the average intake of counterfactual scenarios is equivalent to that of the standard deviations to the average intake at baseline | Prior literature for dietary risk factor models (11)(12). |
| **Comparative risk assessment** | The theoretical minimum risk exposure level for ultra-processed foods consumption is zero | Prior literature have not reported an association of ultra-processed foods and beneficial health outcomes (13) |
|  | All beneficial effects on health are related to reducing the consumption of ultra-processed foods. | Conservatively, we did not consider potential benefits on CVD mortality of replacing ultra-processed foods with fresh and minimally processed foods, increasing fibre, fruit, and vegetable intake, for example. |
|  | The risk of CVD mortality follows a log-linear dose-response to the contribution of ultra-processed foods to total energy intake. | Consistent with previous validated risk assessment models with other dietary risk factors (7)(9). |
|  | The reduction in ultra-processed food consumption and benefits on health and CVD events are assumed to be concurrent. | Consistent with previous macrosimulation studies for other dietary risk factors (14)^,^(12). |
|  |  |  |

**Supplementary Table S8. Relative risks at each 10% of UPD intake estimated according to the contribution of ultra-processed foods to total energy intake (%) by age-group.**

**CHD**

|  | **0%** | **10%** | **20%** | **30%** | **40%** | **50%** | **60%** | **70%** | **80%** | **90%** | **100%** |
| --- | --- | --- | --- | --- | --- | --- | --- | --- | --- | --- | --- |
| **35-39y** | 1.000 | 1.124 | 1.263 | 1.420 | 1.596 | 1.794 | 2.017 | 2.267 | 2.548 | 2.864 | 3.219 |
| **40-44y** | 1.000 | 1.124 | 1.263 | 1.420 | 1.596 | 1.794 | 2.017 | 2.267 | 2.548 | 2.864 | 3.219 |
| **45-49y** | 1.000 | 1.124 | 1.263 | 1.420 | 1.596 | 1.794 | 2.017 | 2.267 | 2.548 | 2.864 | 3.219 |
| **50-54y** | 1.000 | 1.124 | 1.263 | 1.420 | 1.596 | 1.794 | 2.017 | 2.267 | 2.548 | 2.864 | 3.219 |
| **55-59y** | 1.000 | 1.124 | 1.263 | 1.420 | 1.596 | 1.794 | 2.017 | 2.267 | 2.548 | 2.864 | 3.219 |
| **60-64y** | 1.000 | 1.124 | 1.263 | 1.420 | 1.596 | 1.794 | 2.017 | 2.267 | 2.548 | 2.864 | 3.219 |
| **65-69y** | 1.000 | 1.124 | 1.263 | 1.420 | 1.596 | 1.794 | 2.017 | 2.267 | 2.548 | 2.864 | 3.219 |

**Stroke**

|  | **0%** | **10%** | **20%** | **30%** | **40%** | **50%** | **60%** | **70%** | **80%** | **90%** | **100%** |
| --- | --- | --- | --- | --- | --- | --- | --- | --- | --- | --- | --- |
| **35-39y** | 1.000 | 1.147 | 1.315 | 1.508 | 1.730 | 1.983 | 2.275 | 2.609 | 2.992 | 3.431 | 3.934 |
| **40-44y** | 1.000 | 1.147 | 1.315 | 1.508 | 1.730 | 1.983 | 2.275 | 2.609 | 2.992 | 3.431 | 3.934 |
| **45-49y** | 1.000 | 1.147 | 1.315 | 1.508 | 1.730 | 1.983 | 2.275 | 2.609 | 2.992 | 3.431 | 3.934 |
| **50-54y** | 1.000 | 1.147 | 1.315 | 1.508 | 1.730 | 1.983 | 2.275 | 2.609 | 2.992 | 3.431 | 3.934 |
| **55-59y** | 1.000 | 1.147 | 1.315 | 1.508 | 1.730 | 1.983 | 2.275 | 2.609 | 2.992 | 3.431 | 3.934 |
| **60-64y** | 1.000 | 1.147 | 1.315 | 1.508 | 1.730 | 1.983 | 2.275 | 2.609 | 2.992 | 3.431 | 3.934 |
| **65-69y** | 1.000 | 1.147 | 1.315 | 1.508 | 1.730 | 1.983 | 2.275 | 2.609 | 2.992 | 3.431 | 3.934 |

**Supplementary Figure S1. Relative risks for CHD according to the distribution of UPF intake among Brazilian adults.**


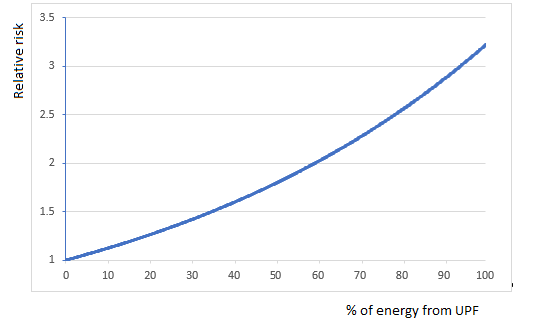


**Supplementary Table S9. Distribution of the contribution of ultra-processed foods to total energy intake (%) at baseline and in the counterfactual modeled scenarios from percentile 5 to over percentile 50.**

|  | **P_5_** | **P_10_** | **P_15_** | **P_20_** | **P_25_** | **P_30_** | **P_35_** | **P_40_** | **P_45_** | **P_50_** | **>=P_50_** |
| --- | --- | --- | --- | --- | --- | --- | --- | --- | --- | --- | --- |
| **Baseline** | 0.00% | 0.17% | 25.96% | 25.77% | 17.32% | 9.97% | 5.40% | 2.86% | 1.51% | 0.80% | 0.01% |
|  |  |  |  |  |  |  |  |  |  |  |  |
| **10% reduction of UPF** | 0.00% | 0.36% | 30.46% | 25.24% | 14.83% | 7.68% | 3.81% | 1.87% | 0.93% | 0.46% | 0.01% |
|  |  |  |  |  |  |  |  |  |  |  |  |
| **20% reduction of UPF** | 0.00% | 0.78% | 34.14% | 23.11% | 11.69% | 5.39% | 2.43% | 1.10% | 0.50% | 0.24% | 0.00% |
|  |  |  |  |  |  |  |  |  |  |  |  |
| **50% reduction in UPF** | 0.15% | 8.91% | 27.59% | 8.36% | 2.34% | 0.67% | 0.20% | 0.06% | 0.02% | 0.01% | 0.00% |
|  |  |  |  |  |  |  |  |  |  |  |  |

**Supplementary Table S10. Estimated** **Population Attributable Factors** **for the total burden of coronary heart disease (CHD) and stroke for the consumption of ultra-processed foods in adults from 30 to 69 years of age in Brazil, 2019.**

|  | **Population Attributable Fraction (PAF)** | |
| --- | --- | --- |
|  | **CHD** | **Stroke** |
| **30-34 years** | 0.163  (0.071 to 0.252) | 0.194  (0.076 to 0.300) |
| **35-39 years** | 0.164  (0.067 to 0.26) | 0.175  (0.068 to 0.272) |
| **40-44 years** | 0.138  (0.056 to 0.214) | 0.170  (0.067 to 0.266) |
| **45-49 years** | 0.158  (0.067 to 0.248) | 0.167  (0.067 to 0.260) |
| **50-54 years** | 0.136  (0.056 to 0.214) | 0.157  (0.064 to 0.244) |
| **55-59 years** | 0.129  (0.053 to 0.204) | 0.146  (0.059 to 0.227) |
| **60-64 years** | 0.115  (0.048 to 0.183) | 0.146  (0.060 to 0.225) |
| **65-69 years** | 0.126  (0.053 to 0.204) | 0.144  (0.059 to 0.222) |
| **Total** | 0.141  (0.059 to 0.222) | 0.162  (0.065 to 0.252) |

**Supplementary Table S11. Estimated deaths from cardiovascular diseases attributable to the consumption of ultra-processed foods disaggregated by age and sex groups in Brazil, 2019.**

|  | **Deaths CVD** | **P2.5** | **P97.5** | **Cases CVD** | **P2.5** | **P97.5** | **DALYs CVD** | **P2.5** | **P97.5** |
| --- | --- | --- | --- | --- | --- | --- | --- | --- | --- |
| Men 15-19y | 62 | 20 | 112 | 601 | 177 | 1,130 | 6,436 | 2,098 | 11,774 |
| Men 20-24y | 104 | 37 | 185 | 880 | 297 | 1,602 | 9,125 | 3,164 | 16,489 |
| Men 25-29y | 146 | 55 | 260 | 1,255 | 456 | 2,268 | 12,142 | 4,480 | 21,848 |
| Men 30-34y | 210 | 77 | 353 | 1,469 | 498 | 2,534 | 17,127 | 6,187 | 28,933 |
| Men 35-39y | 399 | 156 | 693 | 2,036 | 691 | 3,737 | 29,573 | 11,449 | 51,659 |
| Men 40-44y | 585 | 225 | 970 | 2,591 | 899 | 4,480 | 38,118 | 14,552 | 63,487 |
| Men 45-49y | 1,110 | 422 | 1,891 | 4,536 | 1,571 | 7,895 | 65,951 | 25,172 | 112,215 |
| Men 50-54y | 1,576 | 608 | 2,617 | 5,928 | 2,144 | 10,089 | 80,716 | 30,988 | 134,306 |
| Men 55-59y | 2,070 | 790 | 3,469 | 7,602 | 2,796 | 12,910 | 94,182 | 35,869 | 157,970 |
| Men 60-64y | 2,407 | 885 | 4,001 | 7,643 | 2,727 | 12,835 | 90,674 | 33,097 | 151,137 |
| Men 65-69y | 3,055 | 1,138 | 5,227 | 8,612 | 3,154 | 14,839 | 98,046 | 36,622 | 167,594 |
| Men 70-74y | 3,248 | 1,157 | 5,680 | 7,628 | 2,700 | 13,366 | 89,774 | 32,103 | 156,837 |
| Men 75-79y | 2,916 | 1,048 | 5,182 | 5,073 | 1,825 | 9,009 | 62,471 | 22,594 | 110,771 |
| Men 80y+ | 5,587 | 2,039 | 10,225 | 6,446 | 2,352 | 11,798 | 77,502 | 29,023 | 141,436 |
|  |  |  |  |  |  |  |  |  |  |
| Women 15-19y | 37 | 12 | 67 | 754 | 211 | 1,425 | 4,724 | 1,372 | 8,840 |
| Women 20-24y | 70 | 22 | 126 | 1,038 | 319 | 1,896 | 6,859 | 2,071 | 12,602 |
| Women 25-29y | 88 | 29 | 157 | 1,238 | 395 | 2,214 | 7,871 | 2,426 | 14,214 |
| Women 30-34y | 142 | 48 | 249 | 1,699 | 527 | 3,042 | 13,107 | 4,187 | 23,278 |
| Women 35-39y | 257 | 85 | 446 | 2,097 | 626 | 3,753 | 20,781 | 6,819 | 36,278 |
| Women 40-44y | 441 | 151 | 758 | 2,845 | 866 | 5,062 | 31,705 | 10,610 | 55,008 |
| Women 45-49y | 722 | 249 | 1,239 | 3,752 | 1,184 | 6,604 | 43,381 | 15,022 | 74,388 |
| Women 50-54y | 982 | 347 | 1,668 | 4,845 | 1,599 | 8,387 | 53,160 | 18,718 | 90,336 |
| Women 55-59y | 1,260 | 461 | 2,104 | 5,673 | 1,949 | 9,654 | 60,445 | 21,744 | 101,526 |
| Women 60-64y | 1,778 | 649 | 2,977 | 6,629 | 2,314 | 11,245 | 71,467 | 26,046 | 119,713 |
| Women 65-69y | 2,208 | 805 | 3,692 | 6,904 | 2,436 | 11,659 | 74,587 | 27,196 | 124,767 |
| Women 70-74y | 2,622 | 939 | 4,414 | 6,726 | 2,361 | 11,389 | 76,454 | 27,553 | 128,479 |
| Women 75-79y | 2,898 | 1,024 | 4,879 | 5,412 | 1,890 | 9,142 | 65,855 | 23,511 | 110,581 |
| Women 80y+ | 10,502 | 3,604 | 18,007 | 12,333 | 4,102 | 21,339 | 147,546 | 51,893 | 251,230 |

**Supplementary Table S12. Estimated deaths from cardiovascular diseases averted if UPF intake was reduced by 10% disaggregated by age and sex groups in Brazil, 2019.**

|  | **Deaths CVD** | **P2.5** | **P97.5** | **Cases CVD** | **P2.5** | **P97.5** | **DALYs CVD** | **P2.5** | **P97.5** |
| --- | --- | --- | --- | --- | --- | --- | --- | --- | --- |
| Men 15-19y | 7 | 2 | 20 | 70 | 18 | 198 | 745 | 195 | 2,058 |
| Men 20-24y | 12 | 2 | 34 | 99 | 19 | 287 | 1,034 | 194 | 2,974 |
| Men 25-29y | 17 | 5 | 38 | 143 | 42 | 329 | 1,394 | 408 | 3,186 |
| Men 30-34y | 23 | 8 | 52 | 165 | 52 | 376 | 1,929 | 664 | 4,298 |
| Men 35-39y | 44 | 14 | 117 | 224 | 60 | 654 | 3,274 | 1,038 | 8,799 |
| Men 40-44y | 63 | 21 | 133 | 277 | 71 | 615 | 4,103 | 1,342 | 8,735 |
| Men 45-49y | 119 | 43 | 312 | 488 | 171 | 1,278 | 7,115 | 2,575 | 18,601 |
| Men 50-54y | 171 | 51 | 410 | 641 | 168 | 1,617 | 8,808 | 2,603 | 21,175 |
| Men 55-59y | 220 | 86 | 450 | 806 | 300 | 1,668 | 10,081 | 3,932 | 20,596 |
| Men 60-64y | 258 | 96 | 596 | 818 | 284 | 1,868 | 9,776 | 3,588 | 22,528 |
| Men 65-69y | 320 | 104 | 826 | 902 | 286 | 2,340 | 10,337 | 3,357 | 26,653 |
| Men 70-74y | 347 | 86 | 882 | 815 | 202 | 2,073 | 9,646 | 2,403 | 24,506 |
| Men 75-79y | 301 | 107 | 831 | 523 | 185 | 1,446 | 6,488 | 2,271 | 17,936 |
| Men 80y+ | 582 | 53 | 2,003 | 671 | 62 | 2,311 | 8,133 | 680 | 28,024 |
|  |  |  |  |  |  |  |  |  |  |
| Women 15-19y | 4 | 1 | 9 | 89 | 20 | 200 | 559 | 133 | 1,241 |
| Women 20-24y | 8 | 2 | 17 | 121 | 31 | 260 | 805 | 202 | 1,738 |
| Women 25-29y | 10 | 3 | 21 | 142 | 39 | 294 | 909 | 236 | 1,904 |
| Women 30-34y | 16 | 5 | 32 | 194 | 51 | 400 | 1,501 | 409 | 3,060 |
| Women 35-39y | 29 | 8 | 57 | 236 | 60 | 482 | 2,344 | 666 | 4,639 |
| Women 40-44y | 49 | 15 | 96 | 319 | 83 | 648 | 3,570 | 1,039 | 7,006 |
| Women 45-49y | 81 | 24 | 156 | 420 | 114 | 839 | 4,872 | 1,480 | 9,400 |
| Women 50-54y | 109 | 34 | 207 | 538 | 154 | 1,048 | 5,925 | 1,843 | 11,262 |
| Women 55-59y | 138 | 45 | 256 | 623 | 189 | 1,182 | 6,672 | 2,139 | 12,433 |
| Women 60-64y | 196 | 64 | 363 | 729 | 225 | 1,379 | 7,903 | 2,571 | 14,694 |
| Women 65-69y | 242 | 79 | 449 | 758 | 237 | 1,423 | 8,229 | 2,681 | 15,255 |
| Women 70-74y | 288 | 92 | 540 | 740 | 230 | 1,395 | 8,452 | 2,713 | 15,777 |
| Women 75-79y | 316 | 99 | 590 | 591 | 183 | 1,107 | 7,228 | 2,301 | 13,438 |
| Women 80y+ | 1,169 | 352 | 2,252 | 1,373 | 398 | 2,677 | 16,497 | 5,117 | 31,480 |

**Supplementary Table S13. Estimated deaths from cardiovascular diseases averted if UPF intake was reduced by 20% disaggregated by age and sex groups in Brazil, 2019.**

|  | **Deaths CVD** | **P2.5** | **P97.5** | **Cases CVD** | **P2.5** | **P97.5** | **DALYs CVD** | **P2.5** | **P97.5** |
| --- | --- | --- | --- | --- | --- | --- | --- | --- | --- |
| Men 15-19y | 14 | 5 | 30 | 138 | 39 | 300 | 1,483 | 481 | 3,191 |
| Men 20-24y | 23 | 8 | 50 | 197 | 62 | 431 | 2,046 | 661 | 4,450 |
| Men 25-29y | 32 | 10 | 70 | 273 | 83 | 605 | 2,650 | 813 | 5,877 |
| Men 30-34y | 47 | 16 | 92 | 328 | 101 | 651 | 3,847 | 1,279 | 7,556 |
| Men 35-39y | 85 | 33 | 182 | 437 | 149 | 1,017 | 6,354 | 2,408 | 13,651 |
| Men 40-44y | 124 | 50 | 249 | 551 | 200 | 1,152 | 8,149 | 3,257 | 16,405 |
| Men 45-49y | 239 | 91 | 520 | 977 | 350 | 2,135 | 14,238 | 5,460 | 31,045 |
| Men 50-54y | 338 | 113 | 629 | 1,274 | 389 | 2,435 | 17,410 | 5,777 | 32,451 |
| Men 55-59y | 445 | 129 | 939 | 1,636 | 452 | 3,489 | 20,365 | 5,872 | 42,990 |
| Men 60-64y | 501 | 172 | 1,091 | 1,596 | 522 | 3,506 | 19,022 | 6,456 | 41,481 |
| Men 65-69y | 639 | 227 | 1,228 | 1,801 | 624 | 3,491 | 20,641 | 7,377 | 39,576 |
| Men 70-74y | 674 | 215 | 1,467 | 1,581 | 501 | 3,442 | 18,735 | 5,990 | 40,818 |
| Men 75-79y | 612 | 197 | 1,476 | 1,064 | 343 | 2,567 | 13,195 | 4,267 | 31,832 |
| Men 80y+ | 1,149 | 334 | 3,299 | 1,325 | 385 | 3,807 | 16,095 | 4,731 | 45,209 |
|  |  |  |  |  |  |  |  |  |  |
| Women 15-19y | 9 | 2 | 18 | 175 | 42 | 378 | 1,100 | 276 | 2,346 |
| Women 20-24y | 16 | 5 | 33 | 239 | 65 | 493 | 1,586 | 422 | 3,293 |
| Women 25-29y | 20 | 6 | 40 | 281 | 81 | 561 | 1,794 | 493 | 3,622 |
| Women 30-34y | 32 | 10 | 62 | 383 | 106 | 762 | 2,964 | 858 | 5,839 |
| Women 35-39y | 57 | 18 | 109 | 466 | 123 | 921 | 4,639 | 1,399 | 8,889 |
| Women 40-44y | 98 | 31 | 184 | 631 | 171 | 1,239 | 7,069 | 2,190 | 13,439 |
| Women 45-49y | 160 | 52 | 300 | 830 | 237 | 1,606 | 9,655 | 3,136 | 18,067 |
| Women 50-54y | 216 | 72 | 399 | 1,065 | 324 | 2,012 | 11,754 | 3,912 | 21,692 |
| Women 55-59y | 275 | 97 | 495 | 1,236 | 399 | 2,278 | 13,253 | 4,549 | 24,016 |
| Women 60-64y | 389 | 136 | 702 | 1,448 | 477 | 2,660 | 15,702 | 5,481 | 28,386 |
| Women 65-69y | 481 | 168 | 868 | 1,504 | 503 | 2,746 | 16,352 | 5,715 | 29,485 |
| Women 70-74y | 573 | 195 | 1,042 | 1,468 | 487 | 2,691 | 16,788 | 5,774 | 30,466 |
| Women 75-79y | 628 | 211 | 1,140 | 1,173 | 387 | 2,138 | 14,364 | 4,887 | 25,977 |
| Women 80y+ | 2,317 | 744 | 4,330 | 2,720 | 837 | 5,142 | 32,719 | 10,864 | 60,594 |

**Supplementary Table S14. Estimated deaths from cardiovascular diseases averted if UPF intake was reduced by 50% disaggregated by age and sex groups in Brazil, 2019.**

|  | **Deaths CVD** | **P2.5** | **P97.5** | **Cases CVD** | **P2.5** | **P97.5** | **DALYs CVD** | **P2.5** | **P97.5** |
| --- | --- | --- | --- | --- | --- | --- | --- | --- | --- |
| Men 15-19y | 33 | 11 | 65 | 322 | 95 | 651 | 3,448 | 1,129 | 6,838 |
| Men 20-24y | 56 | 19 | 111 | 472 | 148 | 959 | 4,909 | 1,585 | 9,902 |
| Men 25-29y | 78 | 28 | 149 | 672 | 232 | 1,306 | 6,519 | 2,290 | 12,596 |
| Men 30-34y | 110 | 39 | 200 | 773 | 252 | 1,417 | 9,045 | 3,150 | 16,443 |
| Men 35-39y | 208 | 74 | 398 | 1,065 | 344 | 2,088 | 15,510 | 5,468 | 29,759 |
| Men 40-44y | 304 | 113 | 529 | 1,353 | 452 | 2,432 | 19,941 | 7,347 | 34,806 |
| Men 45-49y | 578 | 207 | 1,122 | 2,368 | 773 | 4,634 | 34,482 | 12,443 | 66,956 |
| Men 50-54y | 813 | 317 | 1,509 | 3,060 | 1,115 | 5,855 | 41,861 | 16,251 | 77,888 |
| Men 55-59y | 1,060 | 396 | 1,894 | 3,900 | 1,390 | 7,039 | 48,532 | 18,093 | 86,724 |
| Men 60-64y | 1,243 | 482 | 2,190 | 3,953 | 1,472 | 7,061 | 47,165 | 18,138 | 83,373 |
| Men 65-69y | 1,561 | 611 | 2,767 | 4,402 | 1,681 | 7,843 | 50,389 | 19,803 | 89,271 |
| Men 70-74y | 1,677 | 626 | 3,128 | 3,939 | 1,460 | 7,367 | 46,634 | 17,480 | 86,804 |
| Men 75-79y | 1,505 | 518 | 2,878 | 2,618 | 902 | 5,005 | 32,457 | 11,297 | 61,981 |
| Men 80y+ | 2,836 | 947 | 5,932 | 3,272 | 1,093 | 6,845 | 39,542 | 13,475 | 82,304 |
|  |  |  |  |  |  |  |  |  |  |
| Women 15-19y | 20 | 6 | 39 | 409 | 110 | 823 | 2,570 | 717 | 5,126 |
| Women 20-24y | 37 | 11 | 72 | 560 | 167 | 1,089 | 3,712 | 1,082 | 7,255 |
| Women 25-29y | 47 | 15 | 89 | 661 | 205 | 1,253 | 4,219 | 1,263 | 8,067 |
| Women 30-34y | 75 | 25 | 140 | 903 | 273 | 1,706 | 6,988 | 2,179 | 13,117 |
| Women 35-39y | 135 | 44 | 247 | 1,105 | 322 | 2,073 | 10,987 | 3,539 | 20,191 |
| Women 40-44y | 232 | 78 | 421 | 1,497 | 445 | 2,795 | 16,747 | 5,510 | 30,610 |
| Women 45-49y | 379 | 129 | 686 | 1,971 | 609 | 3,642 | 22,875 | 7,810 | 41,362 |
| Women 50-54y | 513 | 179 | 918 | 2,534 | 823 | 4,602 | 27,919 | 9,719 | 49,949 |
| Women 55-59y | 654 | 238 | 1,150 | 2,949 | 1,002 | 5,260 | 31,578 | 11,271 | 55,723 |
| Women 60-64y | 925 | 335 | 1,630 | 3,449 | 1,192 | 6,144 | 37,376 | 13,518 | 65,884 |
| Women 65-69y | 1,147 | 415 | 2,017 | 3,587 | 1,254 | 6,360 | 38,960 | 14,106 | 68,526 |
| Women 70-74y | 1,364 | 484 | 2,414 | 3,499 | 1,216 | 6,222 | 39,980 | 14,291 | 70,641 |
| Women 75-79y | 1,501 | 526 | 2,649 | 2,804 | 971 | 4,962 | 34,320 | 12,167 | 60,413 |
| Women 80y+ | 5,499 | 1,861 | 9,926 | 6,460 | 2,114 | 11,747 | 77,616 | 26,967 | 139,275 |

**Supplementary** **Figure 2. Sensitivity analysis scenarios compared to the primary estimates for adults from 30 to 69 years of age in Brazil, 2019.**

Grey bars indicate central estimates of n=1,000 simulations and error bars represent 95% uncertainty intervals around the central estimates. Values inside bars indicate the central estimate as a proportion of the central estimate of the primary analysis.

RR: relative risk; Max: maximum; Min: minimum

**Supplementary Table S15.** **Application of the STREAMS-P tool**(15) **for assessing and reporting how methodological decisions were made in the development and validation of the model.**

| **Section** | Item should be present |  |
| --- | --- | --- |
| **Title and abstract** | Risk/s factor/s under study | Page 1-2 |
|  | Country or area under study | Page 1-2 |
|  | Age range under study | Yes |
|  | Information about the methodology | Page 1-2 |
| **Introduction/ Objectives** | Risk(s) factor(s) under study | Page 1-2 |
|  | Key aspects of the population: location and year of study | Page 1-2 |
| **Methods** | |  |
| Methodology | Main characteristics of applied method | Page 3-5 |
| Observed mortality | Cause(s) and the International Classification of Diseases (ICD) Code | Yes |
|  | Causal, causal/suggested, all-causes approach | Page 4 |
|  | Underlying/contributory causes of death | No |
|  | Age range studied | Yes |
|  | Year/s of mortality data | Yes |
|  | Data sources (whether registry-based or not) | Page 3 |
|  | Include the proportion of deaths in the category known as "garbage codes" | Yes |
| Prevalence | Data source (representativeness, response rate and year of study) | Page 3 |
|  | Definition of the categories of exposure | No |
|  | Self-reported vs. objective measures | Page 3 |
|  | Describes choice of groupings by category of exposure | No |
|  | Age-dependent categories of exposure | Page 3 |
|  | Considers intensity of exposure | Yes |
|  | Considers duration of exposure | No |
|  | Uses a correction factor (if neccesary) | NA |
| Risks | Data source, including sample size, place and date of study | Page 3 |
|  | Age-group specific risks (if necessary) | No |
|  | The impact of the adjustment for potential confounders | No |
| **Results** | Observed mortality figures | Page 5 |
|  | Attributed mortality figures taking into consideration selected groupings | Page 5-6 |
|  | Population attributable fractions in selected groupings | Supp. Material |
|  | Prevalence and their precision (eg, 95% confidence intervals) as handled in the analyses. | Yes |
|  | Risk values and their precision (eg, 95% confidence intervals) as handled in the analyses. | Yes |
|  | Attributed mortality precision (eg, 95% confidence intervals) | Yes |
|  | Sensitivity analysis | Yes (page 6 and Supplementary Materials) |
| **Discussion** | Statement on prevalence employed | Yes |
|  | Prevalence correction (if applied) | NA |
|  | Statement on the risks employed | Yes |
|  | Statement on the observed mortality employed and its validity | Yes |
|  | Statement on the strength of evidence regarding the exposure-risk association | Page 4 |

References

1. IBGE. Pesquisa de orçamentos familiares : POF 2017-2018 : análise do consumo alimentar pessoal no Brasil [Internet]. Rio de Janeiro, Brazil; 2020. 114 p. Available from: https://biblioteca.ibge.gov.br/visualizacao/livros/liv101742.pdf

2. Levy RB, Andrade GC, Cruz GL da, Rauber F, Louzada ML da C, Claro RM, et al. Três décadas da disponibilidade domiciliar de alimentos segundo a NOVA – Brasil, 1987–2018. Rev Saude Publica [Internet]. 2022;56:Epub 03. Available from: http://dx.doi.org/10.11606/s1518-8787.2022056004570

3. Monteiro CA, Cannon G, Levy RB, Moubarac J-C, Louzada MLC, Rauber F, et al. Ultra-processed foods: What they are and how to identify them. Public Health Nutr [Internet]. 2019;22(5):936–41. Available from: https://doi.org/10.1017/S1368980018003762

4. Nilson EA, Ferrari G, Louzada MLC, Levy RB, Monteiro CA, Rezende LFM. Modeling of attributable deaths to the consumption of ultra-processed foods in Brazil (Estudo de modelagem de mortes atribuíveis ao consumo de alimentos ultraprocessados no Brasil). In: INFORMAS Regional eSymposium for Latin America and the Caribbean, 2022 [Internet]. 2022. Available from: https://www.informas.org/esymposium-2021-abstracts-presented/

5. Pagliai G, Dinu M, Madarena MP, Bonaccio M, Iacoviello L, Sofi F. Consumption of ultra-processed foods and health status: a systematic review and meta-analysis. Br J Nutr [Internet]. 2020;125(3):308–18. Available from: https://doi.org/10.1017/s0007114520002688

6. Schnabel L, Kesse-Guyot E, Allès B, Touvier M, Srour B, Hercberg S, et al. Association between Ultraprocessed Food Consumption and Risk of Mortality among Middle-aged Adults in France. JAMA Intern Med [Internet]. 2019;179(4):490–8. Available from: https://jamanetwork.com/journals/jamainternalmedicine/article-abstract/2723626

7. Scarborough P, Harrington RA, Mizdrak A, Zhou LM, Doherty A. The Preventable Risk Integrated ModEl and Its Use to Estimate the Health Impact of Public Health Policy Scenarios. Scientifica (Cairo) [Internet]. 2014;2014:748750. Available from: https://doi.org/10.1155/2014/748750

8. Ministério da Saúde. SIM - Mortality Information System [Internet]. 2017. Available from: http://tabnet.datasus.gov.br/cgi/deftohtm.exe?sim/cnv/obt10uf.def

9. IHME. Global burden of disease 2019 [Internet]. 2020. Available from: https://vizhub.healthdata.org/gbd-compare

10. IBGE. Projeção da população do Brasil e das Unidades da Federaçã [Internet]. Projeção da população do Brasil e das Unidades da Federaçã. 2019. Available from: https://www.ibge.gov.br/apps/populacao/projecao/

11. Vega-Solano J, Blanco-Metzler A, Madriz-Morales K, Fernandes-Nilson E-A, Labonté ME. Impact of salt intake reduction on CVD mortality in Costa Rica: A scenario modelling study. PLoS One [Internet]. 2021 [cited 2021 Jan 12];16(1):e0245388. Available from: https://dx.plos.org/10.1371/journal.pone.0245388

12. Nilson EAF, Metzler AB, Labonte M-E, Jaime PC. Modelling the effect of compliance with WHO salt recommendations on cardiovascular disease mortality and costs in Brazil. PLoS One [Internet]. 2020;15(7):e0235514. Available from: https://doi.org/10.1371/journal.pone.0235514

13. Elizabeth L, Machado P, Zinöcker M, Baker P, Lawrence M. Ultra-Processed Foods and Health Outcomes: A Narrative Review. Nutrients [Internet]. 2020;12(7):1955. Available from: doi:10.3390/nu12071955

14. Trieu K, Coyle DH, Afshin A, Neal B, Marklund M, Wu JHY. The estimated health impact of sodium reduction through food reformulation in Australia: A modeling study. Adams J, editor. PLOS Med [Internet]. 2021 Oct 26 [cited 2021 Oct 30];18(10):e1003806. Available from: https://journals.plos.org/plosmedicine/article?id=10.1371/journal.pmed.1003806

15. Pérez-Ríos M, Rey-Brandariz J, Galán I, Fernández E, Montes A, Santiago-Pérez MI, et al. Methodological guidelines for the estimation of attributable mortality using a prevalence-based method: The STREAMS-P tool. J Clin Epidemiol [Internet]. 2022 Jul 1 [cited 2022 May 31];147:101–10. Available from: http://www.jclinepi.com/article/S0895435622000774/fulltext
